# A clinical conceptual framework for testing and designing neuromotor rehabilitation programs in individuals with Bainbridge Ropers syndrome: a perspective study

**DOI:** 10.3389/fneur.2026.1692574

**Published:** 2026-04-24

**Authors:** Srivatsav Yaddanapudi, Phuong T. M. Quach, Jana Villanueva, Keith McGregor, Amy Brady, Harshvardhan Singh

**Affiliations:** 1 College of Arts and Sciences, University of Alabama at Birmingham, Birmingham, AL, United States; 2 Department of Physical Therapy, University of Alabama at Birmingham, Birmingham, AL, United States; 3 Department of Clinical and Diagnostic Sciences, University of Alabama at Birmingham, Birmingham, AL, United States; 4 Department of Nuclear Medicine and Molecular Imaging Science, University of Alabama at Birmingham, Birmingham, AL, United States; 5 Human Performance and Nutrition Research Institute, Oklahoma State University, Stillwater, OK, United States

**Keywords:** cerebral palsy, clinical decision making, genetics, rehabilitation, spina bifida

## Abstract

The Additional Sex Combs Like gene (ASXL) encodes a group of transcriptional regulator proteins that bind to chromatin primarily during fetal development. Mutation of the third transcription factor from this gene (ASXL-3) is responsible for the clinical condition of Bainbridge-Ropers syndrome (BRPS). BRPS is classified as a rare disorder and characterized by multiple physiological/ structural/ biomechanical deficits such as hypotonic muscle development, muscle weakness, foot planovarus/valgus deformities, and gait abnormalities. There exists limited evidence-based medical care and rehabilitation regimen for individuals with BRPS. Thus, the health care needs of individuals with BRPS are hugely unmet. The similarity of clinical manifestations of BRPS with other neurodevelopmental disorders of childhood such as cerebral palsy and spina bifida could help us guide toward forming an assessment and management approach for individuals with BRPS. In the following perspective manuscript, we propose an assessment and management plan, for individuals with BRPS. Specifically, our perspective manuscript lays down a preliminary platform to utilize assessment and rehabilitation techniques from other neurological conditions which characteristically share common neuromotor deficits with BRPS to build evidence-based testing and management techniques for individuals with BRPS.

## Introduction

The Additional Sex Combs Like gene (ASXL) is a group of transcriptional regulators that bind to chromatin typically during the development of the fetus. Point mutations of ASXL can occur along three transcription factors: ASXL-1, ASXL-2 and ASXL-3 mutations. ASXL-1 mutation generally results in malignant myeloid diseases such as acute myeloid leukemia (1). ASXL-2 mutation is associated with cancers such as breast cancer, pancreatic cancer and prostate cancer (2). Our paper is chiefly focused on the ASXL-3 mutation which is the loss of function mutations on the ASXL gene resulting in Bainbridge-Ropers syndrome (BRPS). Clinical manifestations of BRPS include hypotonic muscle development, speech difficulties, neuromotor developmental disorders, muscle weakness, gait abnormalities, and physical abnormalities such as foot planovarus/planovalgus deformities (3), and thus, an overall reduced quality of life from a young age (4). While additional clinical manifestations also persist among BRPS, we are solely looking to address physical movement deficits that are associated with the condition and try to improve patients’ movement ability.

At present, the medical and scientific community is aware of 300 known diagnosed patients, justifying the classification of BRPS as a rare disorder (5). Notably, there is a significant lack of information on independent mobility/physical activity/neuromotor rehabilitation in individuals with BRPS. It is well-known that independent mobility/physical activity in individuals with neurological disorders can help improving their quality of life and reducing the burden of health care related costs (6). Thus, establishing a treatment and therapeutic regimen targeted at the maintenance and enhancement of independent mobility in individuals with BRPS is a high priority for the enhanced management of BRPS.

Notably, the musculoskeletal and neural impediments seen in individuals with BRPS are also often observed in other pediatric populations such as those with cerebral palsy (CP) and spina bifida (SB) (7). For example, cerebral palsy is a neurodevelopmental disorder associated with impaired movement, gait imbalances, hypotonic muscle development, postural deviations, and other musculoskeletal deformities (8). Spina bifida, another neurodevelopmental disorder, impairs movement due to the non-optimal development of the spinal cord, and shares similar clinical characteristics with BRPS including hypotonic developments, reduced motor control and postural deviations (9). In fact, there have been reported case studies of BRPS presenting itself as CP in the literature as recently as 2022, with some people presenting with very similar conditions to CP (7). We suggest that well-established interventions known to improve musculoskeletal and neural impediments in ambulatory individuals with CP and SB could inform framing the framework for effective rehabilitation in individuals with BRPS.

CP and SB, while similar in their clinical presentations, do share some differences that are important to note when utilizing their physical therapy and providing clinical care. Firstly, the disorders are caused by development differences in different areas of the central nervous system (10). CP is often caused by developmental complications in the cerebral cortex and motor cortex regions of the brain whereas SB is caused by improper folding of the neural tube that develops into the spinal cord and leads to nerve disruptions from the spinal cord to the peripheral nervous system (11, 12). Due to the different causal factors of the condition alongside difference in neural disruptions associated with SB and CP, it is possible to see differences in neuromotor symptoms of both conditions as well. In order to address this fact, our article focuses on physical therapy that address symptoms common to both CP and SB before applying those to BRPS to try and account for the differences between conditions.

Even with the differences in condition manifestation and possible symptomatic differences due to neural impairments, there are enough similarities between the conditions such as muscle impairment, gait abnormalities, and hypotonic muscle development to justify the use of existing CP and SB physical therapy knowledge in hopes of improving physical therapy for children with BRPS. Thus, the goal of our perspective article is to utilize the available knowledge on assessment and management of children with CP and SB to offer preliminary recommendations for clinical assessment and rehabilitation strategies in individuals with BRPS who may share similar phenotype characteristics with CP/SB, while still acknowledging the need for empirical validation.

### Recommended tests and their requirements

Table 1 displays the tests that are commonly used in assessing neuromotor performance in individuals with CP and SB. These tests are known for their reliability and are clinically used in children with CP. We posit that those tests could be utilized for neuromotor assessment in individuals with BRPS that face motor deficits. We hypothesize that these tests could aid with quantifying the neuromotor deficits in those children with BRPS who share similar neuromotor deficits reported in children with CP. It can be expected that repeated testing, over time, via those tests could create a critical database to understand and capture critical clinically important metrices needed for neuromotor assessment and rehabilitation management in individuals with BRPS. Resources used to conduct these tests are listed in Table 2.

**Table 1:** Commonly used clinical tests in people with cerebral palsy and spina bifida.

Name of test	Standard error of measurement	Minimum clinical significance in patients with CP**
Four Square Step Test	1.34 s	3.71 s
Timed Up-and-Go Test	3.9 s	10.8 s
Step-Up Test	3.69 reps	Varies
Hand Grip Test	0.10 kg	5.0 kg dominant hand 6.2 kg nondominant hand

**Minimum clinical significance displays improvement of symptoms for children with cerebral palsy. All these values are sourced from various publications (15, 29–37).

**Table 2:** Required resources to perform each test (38).

Name of test	Needed equipment	Test time
Four Square Step Test	Stopwatch, four canes	1–3 min
Timed Up-and-Go Test	Armchair and stopwatch	1–3 min
Step-Up Test	10 cm step/block	15 s
Hand Grip Test	Handheld dynamometer	5 min

#### Four Square Step Test

Four Square Step Test is performed to assess neuromotor deficits in a variety of pediatric disorders including CP. During this test, the participant is instructed to move his/her body from one to another square, formed of canes/rods, with both the feet in each square at each step with one lap clockwise and one lap counterclockwise making up a trial. An unsuccessful trial is deemed when the patients either fail to place both feet in the same square before proceeding to move to the next square or get in contact with the canes/rods while moving between the squares. A participant will perform two trials with the best time taken for data. This test assesses postural dynamic balance.

The minimum detectable change of the test is 3.71 s, and the standard error of measurement (SEM) is 1.34 s among a population of independent ambulatory children with CP ages 5–12 years old (13). Four Square Step Test has also demonstrated excellent interrater reliability (ICC = 0.79) for independent ambulatory children and adolescents with cerebral palsy (13). Therefore, we hypothesize that Four Square Step Test could be a valid, reliable, and feasible tool to assess postural balance in individuals with BRPS who might share characteristics with independent ambulatory children and adolescents with cerebral palsy.

#### Timed Up and Go Test

The Timed Up-and-Go Test is performed with the help of a chair and a stopwatch. It is aimed at assessing neuromotor control of the lower extremities, balance and coordination. The timed test begins with the patient starting in a seated position in a chair with armrests that require the participant to stand and ambulate 3 meters, turn around, keep walking and return to sitting. The test is performed twice for reliability.

The minimum detectable change of this test for children with cerebral palsy is 5.29 s with the SEM of 1.9 s (13). From an interrater and construct standpoint, this test is rated as moderate to excellent among 3 raters (0.54, 0.78, and 0.89) respectively (13). Since Timed Up and Go Test mimics a common functional movement of daily life, this test could be used to assess functional/neuromotor performance at baseline as well as functional/neuromotor improvements post any intervention.

#### Lateral Step-Up Test

Lateral Step-Up Test measures lower extremity muscular strength and balance by measuring how many times in a certain time, e.g., 20/30 s, a participant can step up and down on a block of different heights. The user-friendliness, low-technology, high reliability, clinical application, and functional translation make it a popular and feasible test for individuals with neurological conditions such as cerebral palsy (14). In fact, lateral Step-Up Test is highly related to physical activity status in children with CP (14).

This test has been used in children with CP that are able to ambulate in a study conducted by Chrysagis et al. (15). Furthermore, this test is highly reliable (0.89 and 0.92 inter and intra-rater reliability respectively) (14, 16). We believe that the Step-Up Test could be a reliable test to measure muscle strength and balance of individuals with BRPS.

#### Hand Grip Strength Test

Hand Grip Strength Test measures muscle strength of the upper extremities, especially at the hand, wrist and forearm. This test requires a hand-held dynamometer to measure the hand grip in kg applied on the dynamometer. This test has previously shown high test–retest reliability across Gross Motor Function Classification System and Manual Ability Classification System (MACS) among children and adolescents with cerebral palsy (17). The SEM for this test is 1.10 and the minimum detectable change of the test in children with CP is 13.0 (18, 19).

### Current treatments and future developments

Current rehabilitation plans specifically lack neuromotor rehabilitation in individuals with BRPS. For example, much of the recent research has focused on speech impediments, feeding ability and the use of nutritional interventions that have not proven effective (20). Problems related to the head and neck areas are also a major focus of rehabilitation (4). Thus, there is a huge gap in the knowledge of rehabilitation for many movement/neuromotor deficits in individuals with BRPS. Such deficits are clinically highly important because independent ambulation is vital to improving overall health and quality of life in individuals with BRPS.

### Traditional infant physical therapy vs. caring for infants with special needs program (COPCA)-implications for neuromotor rehabilitation in individuals with BRPS

Based on the limited research, there are two frameworks of physical therapy focused on improving motor skills in individuals with neurodevelopmental disorders: traditional infant physical therapy which is more didactic in its teaching and involves training of motor skills and motor control to children, versus COPCA which is uniquely tailored per the child and promotes learning motor control through a controlled environment of exploration (21).

Traditional Infant Physical Therapy is the classical approach using the basic assumptions and clinical statutes associated with infant development (21). The main tenets of this program are centered around the fact that traditional infant physical therapy is guided mainly by physical therapists while the family serves in an assistant-level capacity with the child. Traditional Infant Physical Therapy approach frames the dynamic between the physical therapist, family and child analogous to that of a teacher, the teaching assistants and the student. From a neurodevelopmental standpoint, traditional infant physical therapy is framed around the understanding that developmental changes throughout life provide valuable information to the management of the pathology. Currently, data is severely lacking if the Traditional Infant Physical Therapy can accelerate the neuromotor development in individuals with BRPS.

COPCA has become more prevalent in recent years, in part dictated by the interest in precise rehabilitation. COPCA-related rehabilitation plan is spearheaded by the family with the physical therapist serving as a coach to provide the family with the resources including techniques to accomplish the rehabilitation goals (21). The emphasis of COPCA is the heightened level of family/caregiver autonomy compared to the traditional infant physical therapy program. Because of this individualized approach, COPCA also utilizes a different framework of neurodevelopment to develop points of emphasis known as neuronal group selection theory. Neuronal group selection theory postulates that neurodevelopment is not linear, unlike proposed by classical neurodevelopmental model. In fact, neuronal group selection theory places greater focus on genetics and experiences (21). One of the tenets of neuronal group selection theory is the importance of trial and error in motor development while focusing on functional movement needed to carry out family and social tasks. Notably, in a study comparing different rehabilitation programs (21), COPCA proved to be most effective at enhancing motor skill acquisition and quality of life in children with CP. Although, there is no data on the effects of COPCA on neuromotor development in individuals with BRPS, it can be postulated that COPCA may translate to greater degree of neuromotor rehabilitation in individuals with BRPS. Studies are urgently needed to examine comparative effects of Traditional Infant Physical Therapy vs. COPCA on neuromotor rehabilitation in individuals with BRPS.

### Potential rehabilitation management for individuals with BRPS

There is a severe lack of information on musculoskeletal and neuromotor rehabilitation in individuals with BRPS, specifically for those with BRPS with gross motor delay, low muscle tone and other orthopedic concerns (5). Thus, we posit that well-established rehabilitation strategies focusing on managing muscle weakness, muscle tonicity, balance issues, musculoskeletal deformities, gait deviations, and upper extremity movement, in independent ambulatory individuals with CP could be used to inform neuromotor rehabilitation strategies in individuals with BRPS sharing similar phenotyping with independent ambulatory children with CP.

Resistance/Strength training programs in individuals with CP are well-established to effectively target muscle weakness in individuals with CP. According to a systematic review, a strength training program designed by physical therapists that is approximately 40–50 min per session 3 times a week can provide optimal improvement in overall muscle strength, gross motor function, gait improvement and balance in individuals with CP (22). Specifically, strength training around the knee flexor and extensor muscles showed significant improvement for gait in independent ambulatory individuals with CP however areas such as the ankle flexor, hip adductor and hip extensor showed less significant improvement (22). This knowledge can be used to design a rehabilitation program aimed at improving muscle strength for gait improvement in individuals with BRPS. Thus, based on the clinical similarities between BRPS and CP, starting physical therapy treatment with a similar method of strength training could be an approach that could improve mobility related clinical outcomes for individuals with BRPS.

From a neuromotor rehabilitation standpoint, establishing a regular exercise program and using treatment modalities such as electric, hot/cold temperature application, and biofeedback are all forms of therapies that are effective for individuals with CP and potentially can be used for mitigating motor/neuromotor deficits in individuals with BRPS (23). For example, it is well-established that muscle strength training leads to either no change or a decrease in spasticity in individuals with CP (24). In fact, musculoskeletal deformities such as dynamic foot equinus could benefit from the combined use of ankle/foot orthoses during their strength training programs in individuals with CP (23). Similarly, in individuals with BRPS displaying similar biomechanical deficits, combining the COPCA method and various motor stimulatory techniques such as electrical stimulation during strength training regimen could maximally enhance and individualize the treatment related benefits (21). Such an innovative approach could potentially have high gains for improving neuromotor constructs in individuals with BRPS.

Interestingly, within upper extremity motor deficits in individuals with hemiplegic CP, novel research regarding the use of traditional toys to improve upper extremity movement has been conducted by Galloway and his team (25). This was taken a step further by Srinivasan et al. where they utilized task-based and intensive therapies delivered via customized car-toy mobility devices, addressing upper extremity deficits in individuals with CP (26). Their initial results showed improvement in muscle endurance and increases in how long people were able to participate in moderate to strenuous physical activity (26). In addition, research has already suggested that constraint-induced movement therapy that isolates the affected area in individuals with hemiplegic CP is a valuable physical therapeutic for this condition however it is difficult for clinicians to have the people engaged during the treatment (27). Thus, it could be hypothesized that well-established strategies such as constraint-induced movement therapy or novel approaches such as delivering constraint-induced movement therapy via customized toy-rides could help with upper extremity neuromotor development in individuals with BRPS. With the growth of exergaming, including clinical research into using joystick-led therapy for upper extremity motor deficits, more focus is needed to examine their effectiveness in neuromotor rehabilitation in individuals with BRPS.

### Limitations and challenges to consider

It is important to consider how the support systems around affected individuals with BRPS can use the COPCA framework to assist the child through various rehabilitation strategies to maximally improve their function and quality of life. We know that the most common current practices among families assisting individuals with CP rely on a multidisciplinary team including occupational therapists for maximal effectiveness (28). In addition, they (28) showed that majority of physical therapists and occupational therapists did not address all the areas of body systems in their clients. Thus, it is important for the family to know the therapist-associated limitation/focus areas while constructing a rehabilitation plan for individuals with BRPS.

When embedded within the COPCA framework the strategies mentioned above such as strength training, joystick-led upper extremity therapy, the use of ankle orthoses during strength and resistance training, can be utilized alone or in combination, based on the individual needs of the client. Thus, novel, clinically feasible, and effective ways can be designed for neuromotor rehabilitation in individuals with BRPS.

## Conclusion

Our perspective manuscript applies the concept of a *researcher’s mind, clinician’s heart and implementation approach* to provide a preliminary hypothesis that clinicians can apply well-established assessment and treatment techniques in another related neurological clinical population to inform the neuromotor rehabilitation with limited number of barriers for individuals with BRPS. More specifically, many of the interventions target neuromotor problems that are shared between BRPS and CP such as gait imbalances, hypotonic muscle development, postural deviations, and other musculoskeletal deformities. Knowledge from our perspective manuscript could provide the knowledge to build a platform that can be utilized for creating evidence-based assessment, evaluation, and rehabilitation plan for individuals with BRPS.

## Data Availability

The original contributions presented in the study are included in the article/supplementary material, further inquiries can be directed to the corresponding author/s.
